# PTP1B inhibitor promotes endothelial cell motility by activating the DOCK180/Rac1 pathway

**DOI:** 10.1038/srep24111

**Published:** 2016-04-07

**Authors:** Yuan Wang, Feng Yan, Qing Ye, Xiao Wu, Fan Jiang

**Affiliations:** 1 Key Laboratory of Cardiovascular Remodelling and Function Research (Chinese Ministry of Education and Chinese Ministry of Health) and The State and Shandong Province Joint Key Laboratory of Translational Cardiovascular Medicine, Qilu Hospital, Shandong University, Jinan, Shandong Province, China

## Abstract

Promoting endothelial cell (EC) migration is important not only for therapeutic angiogenesis, but also for accelerating re-endothelialization after vessel injury. Several recent studies have shown that inhibition of protein tyrosine phosphatase 1B (PTP1B) may promote EC migration and angiogenesis by enhancing the vascular endothelial growth factor receptor-2 (VEGFR2) signalling. In the present study, we demonstrated that PTP1B inhibitor could promote EC adhesion, spreading and migration, which were abolished by the inhibitor of Rac1 but not RhoA GTPase. PTP1B inhibitor significantly increased phosphorylation of p130Cas, and the interactions among p130Cas, Crk and DOCK180; whereas the phosphorylation levels of focal adhesion kinase, Src, paxillin, or Vav2 were unchanged. Gene silencing of DOCK180, but not Vav2, abrogated the effects of PTP1B inhibitor on EC motility. The effects of PTP1B inhibitor on EC motility and p130Cas/DOCK180 activation persisted in the presence of the VEGFR2 antagonist. In conclusion, we suggest that stimulation of the DOCK180 pathway represents an alternative mechanism of PTP1B inhibitor-stimulated EC motility, which does not require concomitant VEGFR2 activation as a prerequisite. Therefore, PTP1B inhibitor may be a useful therapeutic strategy for promoting EC migration in cardiovascular patients in which the VEGF/VEGFR functions are compromised.

Migration of the endothelial cells (ECs) is a fundamental biological process that plays central roles in both embryonic blood vessel development (vasculogenesis)^1^ and postnatal angiogenesis^2^,^3^. Increased EC migration is also favourable for re-endothelialization of the denuded luminal surface of injured blood vessels, which is critical to prevent the development of intimal hyperplasia and stenosis following mechanical vessel injuries^4^,^5^. EC migration is coordinated by complex signalling mechanisms, of which those mediated by vascular endothelial growth factor receptors (VEGFRs) and the Rho family small GTPases have critical roles^6^,^7^,^8^. VEGF is one of the most important chemotactic factors that guide the directional movement of endothelial cells^6^. VEGFRs are conventional receptor tyrosine kinases, among which the VEGFR2 has a predominant role in mediating the activation of downstream pathways involved in EC migration^6^,^9^.

Protein tyrosine phosphatase 1B (PTP1B) is a widely-expressed dephosphorylating enzyme with broad biological functions^10^. Both *in vitro* and *in vivo* studies have shown that PTP1B is a crucial negative regulator of the VEGFR2 signalling in EC^11^,^12^. Recent studies demonstrated that inhibition of the PTP1B function could promote EC migration and postnatal angiogenesis under pathological conditions^12^,^13^. Moreover, treatment with PTP1B inhibitor restored hyperglycaemia-induced defects in EC motility^14^. These effects were all ascribed to the enhanced VEGFR2 signalling following PTP1B inhibition^12^,^13^,^14^. However, PTP1B is a versatile enzyme, which may dephosphorylate multiple substrates (apart from the VEGFR2) that are involved in modulating cell migration^15^. Particularly, in patients with cardiovascular disease, the availability and/or functions of VEGF/VEGFR are compromised^16^. Hence, it will be interesting to clarify whether PTP1B inhibition may also affect EC motility in the absence of functional VEGFR2 signalling.

In addition to VEGFR2, PTP1B also affects the phosphorylation status of proteins involved in the integrin signalling pathway^17^,^18^, which is also critical for orchestrating endothelial cell adhesion and migration^7^. Binding of integrins to extracellular matrix triggers auto-phosphorylation on Tyr397 of the non-receptor tyrosine kinase focal adhesion kinase (FAK), which in turn recruits another tyrosine kinase Src. Activation of Src can stimulate the activity of guanine nucleotide exchange factors (GEFs) Vav2 and Tiam1, leading to Rac1 activation^19^. Alternatively, FAK/Src complex can phosphorylate the adaptor protein p130Cas, and phosphorylated p130Cas binds to another adaptor protein Crk, leading to further recruitment and activation of the Rac1 GEF DOCK180^20^,^21^. Disruption of the functions of FAK or DOCK180 compromises cell migration as well as angiogenesis^20^,^21^,^22^,^23^. However, currently it is unclear whether inhibition of PTP1B may also modulate EC motility via the p130Cas/DOCK180 pathway.

Based on these findings, in the present study we tested the hypothesis that pharmacological inhibition of PTP1B might be able to modulate EC motility even in the absence of functional VEGFR2 signalling. We provided first evidence showing that PTP1B inhibitor could stimulate EC motility by promoting DOCK180-dependent Rac1 activation in the absence of VEGFR2 signalling, suggesting that activation of the DOCK180 pathway might represent an alternative mechanism of PTP1B inhibitor-stimulated EC motility.

## Results

### PTP1B inhibitors enhanced EC adhesion and spreading

**Figure 1:**
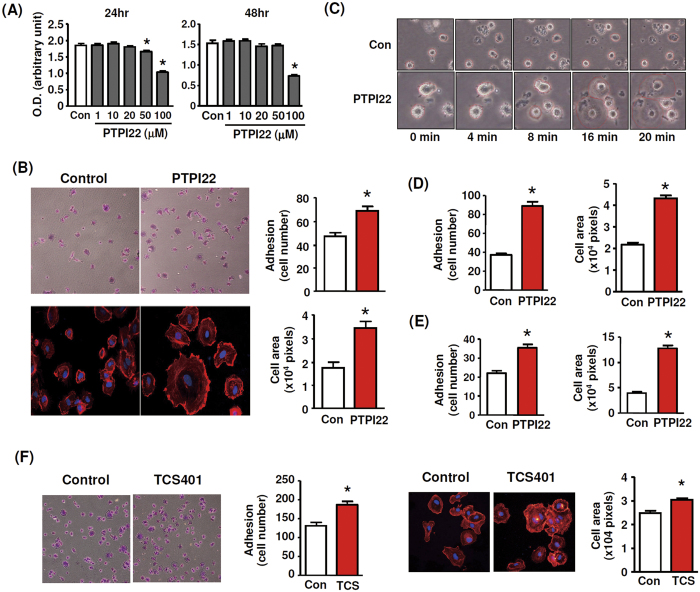
PTP1B inhibitors promoted EC adhesion and spreading. (**A**) Effects of PTPI22 treatment with increasing concentrations on cell viability in telomerase-immortalized human microvascular endothelial (TIME) cells at 24 or 48 hr. (**B**) Effects of PTPI22 treatment (10 μM) on adhesion and spreading of TIME cells on the collagen substratum. Cell adhesion was expressed as the average cell number per high power field. Cell spreading was expressed as the average cell area. (**C**) Time-lapse images of cells without and with PTPI22 treatment recorded under a phase contrast microscope. Cells had been pretreated with PTPI22 for 1 hr before seeding, therefore the size of treated cells at 0 min appeared larger than the control. (**D**) Effects of PTPI22 on adhesion and spreading of primary human umbilical vein endothelial cells (HUVEC) seeded on collagen. (**E**) Effects of PTPI22 on TIME cell adhesion and spreading on an uncoated surface. (**F**) Effects of TCS401 (600 nM) on adhesion and spreading responses in TIME cells. Data were mean ± S.E.M.; *P < 0.05, unpaired *t*-test, *n* = 3–6.

PTP1B Inhibitor XXII (referred to as PTPI22 thereafter) is a cell-permeable selective inhibitor of PTP1B. We first determined potential cytotoxic effects of PTPI22 with increasing concentrations in TIME cells. We found that PTPI22 under 20 μM had no significant cytotoxic effects at 24 or 48 hr (Fig. 1A). In the following experiments, therefore, we used PTPI22 at 10 μM. We demonstrated that PTPI22 treatment significantly enhanced TIME cell adhesion and spreading on the collagen substratum (Fig. 1B). To continuously monitor the dynamic changes of cell motility following PTP1B inhibitor treatment, we recorded digital videos of TIME cells with and without PTPI22 treatment. As shown in Fig. 1C and Supplementary Videos S1 and S2, PTPI22 treatment increased the rate of cell spreading process. To further confirm PTP1B inhibitor effects, we also tested PTPI22 in primary HUVECs. As shown in Fig. 1D, PTPI22 exhibited similar enhancing effects on cell adhesion and spreading in HUVECs. To clarify whether the effects of the PTP1B inhibitor were dependent on specific adhesion substratum, we seeded TIME cells in uncoated culture plates. We found that PTPI22 produced similar increasing effects on EC adhesion and spreading on the uncoated surface (Fig. 1E). In addition, we studied the effects of another small molecule PTP1B inhibitor TCS401. We showed that similar to PTPI22, TCS401 also significantly increased adhesion and spreading responses in TIME cells (Fig. 1F).

### PTP1B inhibitors enhanced EC migration

**Figure 2:**
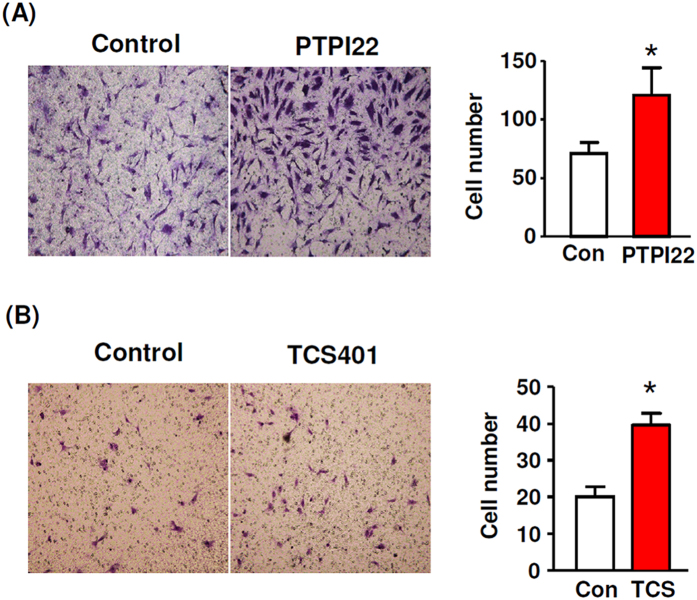
Effects of PTPI22 and TCS401 on serum-stimulated transwell migration of TIME cells assessed by Boyden chamber assay. The quantitative results were expressed as the average cell number per high power field. Data were mean ± S.E.M.; *P < 0.05, unpaired *t*-test, *n* = 4–5.

Next we examined the effects of PTPI22 and TCS401 on EC migration using the transwell assay. We demonstrated that both PTPI22 and TCS401 significantly enhanced TIME cell migration stimulated by serum (Fig. 2).

### The effects of PTP1B inhibitor persisted after VEGFR2 inhibition

**Figure 3:**
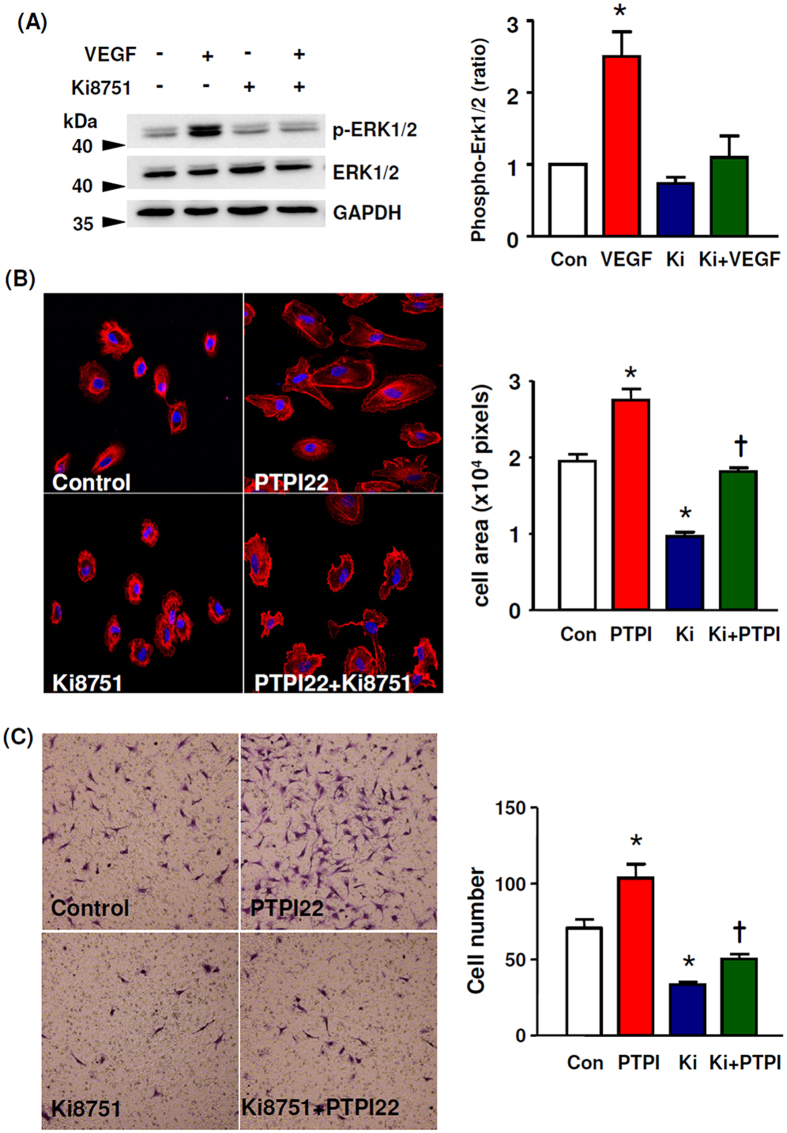
Effects of PTPI22 on cell motility after blockade of VEGFR2 signalling. Cells were pretreated with the VEGFR2 inhibitor Ki8751 (2 nM). (**A**) Western blots and densitometry data showing that Ki8751 abolished VEGF165 (3 ng/ml)-induced phosphorylation of ERK1/2. (**B**) Effects of PTPI22 on cell spreading in the absence or presence of Ki8751. (**C**) Effects of PTPI22 on cell migration in the absence or presence of Ki8751 assessed with Boyden chamber assay. Data were mean ± S.E.M.; *P < 0.05 *vs* control, ^†^*P* < 0.05 *vs* Ki8751, one-way ANOVA, *n* = 4–5.

The above results indicate that PTP1B inhibitors exert stimulatory effects on EC motility. To delineate the associated signalling mechanisms, we pretreated TIME cells with the VEGFR2 inhibitor Ki8751 (2 nM). We showed that Ki8751 at this concentration effectively blocked VEGF-induced ERK1/2 phosphorylation (Fig. 3A). Under the basal condition, Ki8751 significantly reduced the cell spreading response. However, we found that in the presence of Ki8751, PTPI22 still exhibited an enhancing effect on EC spreading (Fig. 3B). This result indicates that PTPI22 can promote EC motility in the absence of VEGFR2 signalling. To confirm this finding, we repeated the experiments using transwell migration assay. We showed that Ki8751 reduced the basal level of cell migration, while PTPI22 still significantly increased EC migration in the presence of Ki8751 (Fig. 3C).

### The effects of PTP1B inhibitor on EC motility were Rac1-dependent

**Figure 4:**
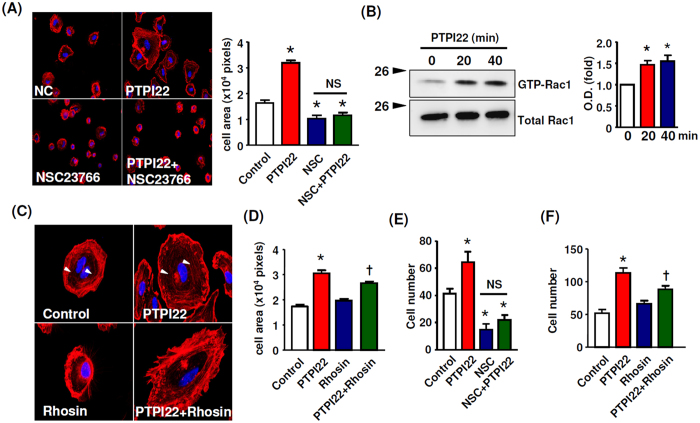
The effects of PTP1B inhibitor on EC motility were dependent on Rac1. (**A**) The effects of PTPI22 on cell spreading in the absence or presence of the Rac1 inhibitor NSC23766 (100 μM). (**B**) GTPase pull-down assay showing that PTPI22 treatment increased the amount of GTP-bound Rac1 in sub-confluent TIME cells. (**C**,**D**) Effects of the Rho inhibitor Rhosin (1 μM) on PTPI22-stimulated cell spreading response. The presence of stress fibres in untreated cells was indicated by white arrowheads; Rhosin decreased the abundance of intracellular stress fibres. (**E**,**F**) Effects of NSC23766 and Rhosin on PTPI22-stimulated EC migration assessed by the Boyden chamber assay. Data were mean ± S.E.M.; *P < 0.05 *vs* control, ^†^*P* < 0.05 *vs* Rhosin, one-way ANOVA, *n* = 3–4. NS, no significance.

Given the pivotal role of Rac small GTPase in mediating cell motility, we examined whether Rac1 activation was involved in the effects of PTP1B inhibitor. TIME cells were pretreated with the Rac1 inhibitor NSC23766 (100 μM). We found that NSC23766 reduced the basal level of cell spreading, and the stimulatory effect of PTPI22 on cell spreading was abolished by NSC23766 (Fig. 4A). Using Rac1 GTPase pull-down assay, we demonstrated that PTPI22 significantly increased Rac1 activation in EC (Fig. 4B). We also demonstrated that PTPI22 could stimulate Rac1 activation in the presence of Ki8751 (Supplementary Fig. S1). To clarify whether Rho GTPase was also implicated in the effects of PTP1B inhibitor, we pretreated cells with the RhoA inhibitor Rhosin (1 μM). In both control and PTPI22-treated cells, Rhosin decreased the abundance of intracellular stress fibres (Fig. 4C). However, Rhosin did not change the stimulatory effects of PTPI22 on EC spreading (Fig. 4C,D). Moreover, we performed transwell migration assays and confirmed that NSC23766 also abolished the stimulatory effect of PTPI22 on EC migration, which was not changed by Rhosin (Fig. 4E,F).

### PTP1B inhibitor did not change the phosphorylation levels of FAK, Src or paxillin in EC

**Figure 5:**
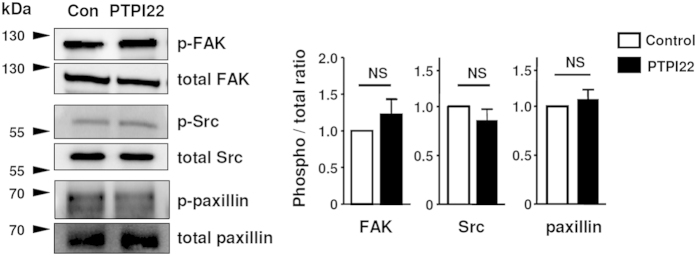
Effects of PTPI22 on the phosphorylation levels of FAK, Src and paxillin in TIME cells. Cell lysates were immunoprecipitated with the anti-phospho tyrosine antibody (P-Tyr-100) followed by western blot detections. The densitometry data were means from 3 independent experiments.

Both of the VEGFR2 and FAK/Src pathways can regulate Rac1 activation and cell motility. In the following experiments, therefore, we investigated whether PTP1B inhibitor affected the FAK/Src signalling pathway. Using immunoprecipitation and western blot, we found that treatment with PTPI22 had no significant effects on the phosphorylation levels of FAK, Src or paxillin (Fig. 5). On the other hand, we demonstrated that Ki8751 reduced the phosphorylation level of FAK in normal ECs (Supplementary Fig. S2).

### The effects of PTP1B inhibitor were dependent on DOCK180

**Figure 6:**
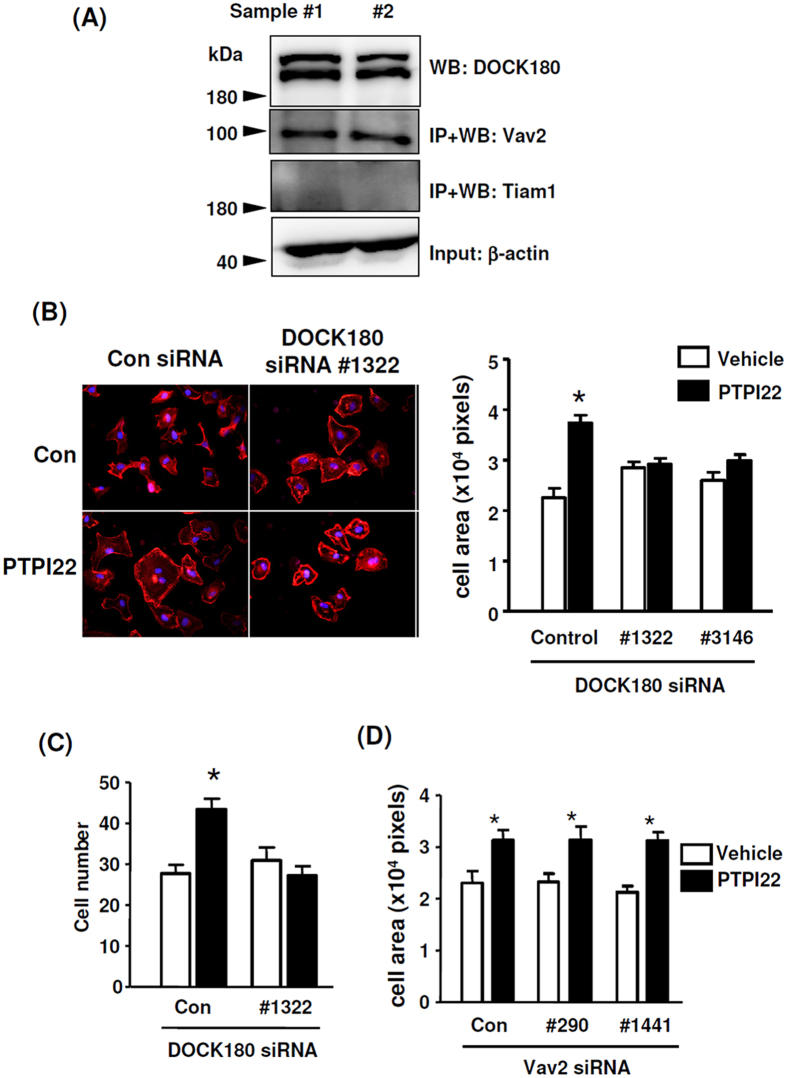
Role of DOCK180 in the effects of PTP1B inhibitor on EC motility. (**A**) Western blots showing the endogenous expression levels of Vav2, Tiam1 and DOCK180. IP, immunoprecipitation; WB, western blot. (**B**,**C**) Effects of gene silencing for DOCK180 with different siRNA constructs on the stimulatory effects of PTPI22 on EC spreading and migration. (**D**) Effects of gene silencing for Vav2 with different siRNA constructs on the stimulatory effect of PTPI22 on EC spreading. Data were mean ± S.E.M.; *P < 0.05 *vs* vehicle control, one-way ANOVA, *n* = 3–5.

In order to define the Rac1 guanine nucleotide exchange factors (GEFs) involved in PTPI22-induced effects, we preformed western blot to detect the expression of endogenous Vav2, Tiam1 and DOCK180. We found that DOCK180 was readily detectable in EC; Vav2 could be detected only after immunoprecipitation. However, Tiam1 was not detectable even with immunoprecipitation, indicating an extremely low expression level in EC (Fig. 6A). Next we performed gene silencing experiments for Vav2 and DOCK180 with siRNA. We found that knocking down of DOCK180 significantly blunted the stimulating effects of PTPI22 on EC spreading and migration (Fig. 6B,C). In contrast, knocking down of Vav2 did not change the effects of PTPI22 (Fig. 6D). To further corroborate that Vav2 was not involved in the PTPI22 effects, we measured the tyrosine phosphorylation level of Vav2. We found that the endogenous level of Vav2 phosphorylation was undetectable in TIME cells, while treating cells with PTPI22 did not increase the level of Vav2 phosphorylation (repeated 2 times).

### PTP1B inhibitor increased p130Cas phosphorylation and p130Cas-Crk-DOCK180 interactions

**Figure 7:**
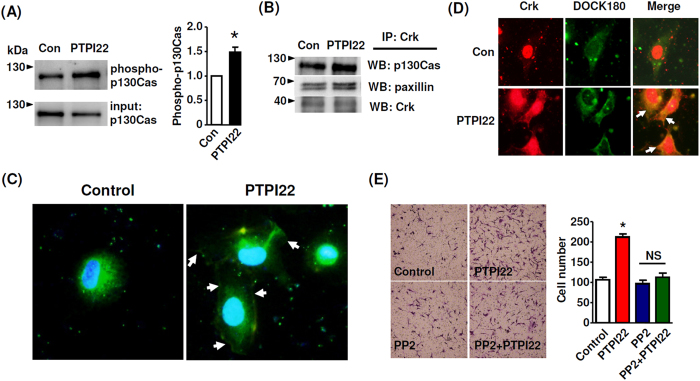
Effects of PTPI22 on p130Cas phosphorylation and interactions among p130Cas, Crk, paxillin and DOCK180. (**A**) The tyrosine phosphorylation level of p130Cas was detected by immunoprecipitation with P-Tyr-100 followed by western blotting. (**B**) Interactions among p130Cas, Crk and paxillin were detected by immunoprecipitation of Crk followed by western blotting detections. (**C**) Confocal microscopy images showing the effect of PTPI22 on intracellular translocation of Crk toward the cell periphery and the plasma membrane (arrows). (**D**) Interaction between Crk and DOCK180 was detected with double immunofluorescence labelling. Treatment with PTPI22 increased co-localisation of Crk and DOCK180 (arrows). (**E**) Effect of the Src inhibitor PP2 (200 nM) on PTPI22-stimulated EC migration. Data were mean ± S.E.M.; *P < 0.05 *vs* control, unpaired *t*-test or one-way ANOVA, *n* = 3–4.

The activity of DOCK180 is controlled by the p130Cas/Crk complex, while p130Cas is a PTP1B substrate^17^. Based on this evidence, we reasoned that PTP1B inhibitor might act to promote the activation of DOCK180. To test this possibility, we first measured the phosphorylation level of p130Cas. As shown in Fig. 7A, PTPI22 treatment significantly increased tyrosine phosphorylation of p130Cas. This effect of PTPI22 was also observed in the presence of Ki8751 (Supplementary Fig. S3). Then we immunoprecipitated Crk, and demonstrated that PTPI22 treatment significantly increased the interaction between Crk and p130Cas (Fig. 7B). PTPI22 also increased binding of Crk with paxillin (Fig. 7B). To detect the interaction between Crk and DOCK180, we labelled them with immunofluorescence. We showed that Crk was highly expressed in the nucleus and in the peri-nuclear region; PTPI22 treatment induced intracellular translocation of Crk toward the cell periphery and the plasma membrane (Fig. 7C), supporting that the Crk function was activated^24^. Moreover, we demonstrated that treatment with PTPI22 increased co-localisation of Crk and DOCK180 (Fig. 7D). To further verify the interactions among p130Cas, Crk and DOCK180, we performed co-immunoprecipitation assays and showed that PTPI22 increased the bindings of DOCK180 with p130Cas and Crk (Supplementary Fig. S4). We confirmed that the effects of PTPI22 were also observable in the presence of Ki8751 (Supplementary Fig. S4). Although we found that the phosphorylation level of Src was not affected by the PTP1B inhibitor, this could not exclude that Src was functionally important in the effects of PTP1B inhibitor^25^. To address this question, we pretreated cells with the Src inhibitor PP2. Interestingly, we found that PP2 blocked the stimulatory effect of PTPI22 on EC migration (Fig. 7E).

## Discussion

PTP1B inhibition may promote EC migration by enhancing VEGFR2 signalling. The major finding of the present study is that PTP1B inhibitor may also stimulate EC motility in the absence of functional VEGFR2 signalling, mainly by promoting p130Cas/DOCK180-dependent Rac1 activation. Although our previous study has demonstrated that PTP1B inhibitor may increase the basal level of VEGFR2 phosphorylation in normal ECs^26^, subsequent results indicate that under the present experimental conditions, the VEGFR2 pathway is constitutively (at least partially) activated, while further stimulation of this pathway by PTP1B inhibitor might not necessarily result in significant increases in certain VEGFR2-mediated EC functions. For example, we showed that Ki8751 significantly reduced EC motility as well as FAK phosphorylation in the absence of VEGF, while stimulation of resting ECs with PTP1B inhibitor exhibited minor effects on cell proliferation (unpublished data). Importantly, we showed that PTP1B inhibitor-stimulated EC motility was totally abrogated by DOCK180 gene silencing. Several lines of evidence support that the p130Cas/DOCK180 pathway may function separately from the VEGFR signalling. Firstly, although p130Cas can be activated by VEGF, in the absence of VEGFR2 activity, p130Cas can also be activated by other upstream pathways^20^,^21^. Secondly, p130Cas is a direct target of PTP1B, and this interaction is critical for normal cell motility functions^17^,^27^. Thirdly, VEGF-induced Rac1 activation in ECs is mainly mediated by Vav2 phosphorylation^28^, while so far there is no evidence showing that DOCK180 is directly stimulated by VEGF in mammalian cells. Moreover, DOCK180 deficiency results in vascular defects but has no impact on VEGF-induced EC migration (reviewed in)^8^, further supporting that DOCK180 is not directly linked to VEGFR signalling. Taken these together, we propose that stimulation of the DOCK180 pathway by PTP1B inhibitor, which does not require concomitant VEGFR2 activation as a prerequisite, may represent an important alternative mechanism of PTP1B inhibitor-stimulated EC motility (see Supplementary Fig. S5).

Endothelial cell migration is a multi-step process involving highly orchestrated cytoskeletal reorganization, plasma membrane protrusion, formation of adhesions at the leading edge, and contraction at the rear of the cell. It is noted that enhanced endothelial cell adhesion and spreading may not necessarily be accompanied by increased cell migration, and the final outcome is variable and appears to be context-dependent^29^,^30^,^31^,^32^. Here we have shown that PTP1B inhibitor enhances adhesion, spreading, as well as migration of EC, and these observations are consistent with previous studies showing that EC migration is regulated in the same fashion as adhesion and/or spreading under different experimental settings^29^,^31^,^32^,^33^. Among the various intracellular mediators of EC motility, Rac1 is a master molecular switch which integrates signals from both growth factor receptors and integrin receptors. Rac1 also coordinates the spatial and temporal pattern of activation/inactivation of Rho-mediated molecular events during cell migration^8^,^34^. In EC, it has been shown that inactivation of Rac1 compromised all of cell spreading, lamellipodia formation, and migration responses^29^,^35^. In accordance with these findings, we have demonstrated that PTPI22 induces Rac1 activation, while the effects of PTPI22 on cell motility are blocked by the specific Rac1 inhibitor NSC23766, suggesting that Rac1 has a pivotal role in mediating the effects of PTP1B inhibitor. As mentioned above, our data support that functional VEGFR2 signalling is not obligatory for PTP1B inhibitor-induced Rac1 activation. In contrast, our results do not support an involvement of Rho, although Rho is also important in maintaining normal cell motility functions^34^.

The FAK/Src pathway has an essential role in mediating Rac1 activation. First, the FAK/Src complex recruits and phosphorylates p130Cas, and tyrosine phosphorylated p130Cas can bind to Crk; the Cas/Crk complex in turn promotes activation of the Rac1 GEF DOCK180^20^. Secondly, Src can phosphorylate and thereby activate the GEF proteins Vav2 and Tiam1, although these effects may not require FAK^19^. There is evidence showing that PTP1B may dephosphorylate the inhibitory tyrosine 527 of Src, leading to an augmented kinase activity^18^. However, we cannot detect changes in Src phosphorylation following PTPI22 treatment. In addition, the phosphorylation level of Vav2 is not affected by PTPI22 either. These data may exclude a major role of the Src/Vav2 pathway. Also, Tiam1 expression appears to be little in endothelial cells. On the other hand, DOCK180 is abundantly expressed in endothelial cells. We have demonstrated that treatment with PTPI22 increases p130Cas phosphorylation and interactions among p130Cas, DOCK180, Crk and paxillin; the critical role of DOCK180 is further corroborated by the observation that gene silencing of DOCK180, but not Vav2, attenuates the effects of PTPI22. Our results are also in line with observations that PTP1B-mediated p130Cas dephosphorylation may compromise adhesion, spreading and migration in non-endothelial cells^17^,^27^,^36^. These results strongly suggest that the PTPI22 effects on EC motility are mediated by p130Cas/DOCK180-dependent Rac1 activation.

The proline-rich regions in the C terminus of PTP1B bind to the SH3 domain of p130Cas, and PTP1B catalyzes p130Cas dephosphorylation^17^. Reversely, the *N*-terminal SH3 domain of p130Cas is responsible for its localization in focal adhesions where it is in juxtaposition with Src, which mediates phosphorylation of the 15 potential substrate domains of p130Cas^37^. It is shown that Src-mediated tyrosine phosphorylation at as few as four of these substrate domains is sufficient to stimulate cell migration^38^. Consistent with this paradigm, we have shown that PTPI22-induced enhancement of EC motility is associated with an increased phosphorylation level of p130Cas. Moreover, the stimulatory effect of PTPI22 is partially inhibited by the Src kinase inhibitor PP2, although PTPI22 does not directly change the phosphorylation level of Src. This finding can be explained by the fact that the steady state of p130Cas phosphorylation is subject to the dual control by both Src and PTP1B. Loss of the constitutive Src activity may mask the role of PTP1B by rendering most of p130Cas in a non-phosphorylated state.

In summary, PTP1B inhibitor may promote EC motility via multiple mechanisms; in addition to enhancing VEGFR2 signalling, we provide evidence showing that activation of the p130Cas/DOCK180/Rac1 pathway may represent an alternative mechanism of PTP1B inhibitor-stimulated EC motility in the absence of functional VEGFR2 signalling. Small molecule PTP1B inhibitors are currently under development for the treatment of type 2 diabetes. Clarification of the mechanisms of PTP1B inhibitor-stimulated EC migration will be of scientific significance not only for modulating postnatal angiogenesis, but also for accelerating the re-endothelialization process after blood vessel injuries, and engineering of functional artificial blood vessels. Our data support that PTP1B inhibitor may represent a useful therapeutic strategy for promoting EC migration in cardiovascular patients in which the VEGF/VEGFR functions are compromised.

## Materials and methods

### Reagents

3-(3,5-dibromo-4-hydroxybenzoyl)-2-ethyl-N-[4-(1,3-thiazol-2-ylsulfamoyl)phenyl]-1-benzofuran-6-sulfonamide (PTP Inhibitor XXII), Rhosin, and NSC23766, PP2, and Ki8751 were all purchased form Merck Millipore (Darmstadt, Germany). TCS401 was purchased from Tocris Bioscience (Bristol, UK).

### Cell culture

Human umbilical vein ECs (HUVECs) and telomerase-immortalized human microvascular endothelial (TIME) cells were purchased from the American Type Culture Collection (ATCC, Rockville, MD, USA). Cells were maintained in complete ECM medium (Catalogue #1001, ScienCell, Carlsbad, CA, USA) supplemented with 5% foetal bovine serum (FBS), the Endothelial Cell Growth Supplement, penicillin (100 U/ml) and streptomycin (100 μg/ml) as used before^39^.

### Transwell cell migration assay

Transwell migration assay was performed using Boyden chambers as described previously^40^. Briefly, cells were trypsinized and resuspended in serum-free medium, and 10^5^ cells were seeded in the upper well. Serum was added to the lower chamber to a final concentration of 1% as a chemotactic factor. After 6 hr of incubation, cells migrating across the membrane were fixed in cold methanol and stained with crystal violet. For each membrane, 5–10 random high power (400×) fields were photographed under a light microscope, and the number of cells was counted. All experiments were repeated at least for 3 times. Drugs or vehicle were added to both of the upper and lower chambers.

### Cell adhesion assay

Cells were pretreated with serum free medium containing vehicle or drugs for 1 hr. Then cells were trypsinized and seeded in 24-well plates in serum-free medium containing the same treatment agent. After 30 min, wells were washed with PBS and fix with cold methanol. Cells were stained with 1% crystal violet. For each well, 5–10 random high power fields were photographed and the number of attached cells counted.

### Cell spreading assay

Cells were pretreated with drugs or vehicle in serum-free medium for 1 hr. Then the cells were replated in 8-well Lab-Tek II chamber slides (Thermo Scientific, Waltham, MA, USA) and further incubated with the same treatment agent for 1 hr. Cells were fixed, stained with Rhodamine phalloidin (from Cytoskeleton, Denver, CO, USA), counterstained with DAPI, and observed under a confocal microscope (Model LSM710, Zeiss, Jena, Germany). The spreading response was evaluated by measuring the average cell area using Image-Pro Plus 6.0 software (Media Cybernetics, Rockville, MD, USA). For each experiment, 150–200 cells from different random fields were analyzed. To monitor the cell motility behaviour in real-time, TIME cells were pretreated with PTP1B inhibitor or vehicle for 1 hr, harvested and re-suspended in serum free medium and seeded in 6-well plates at a concentration of 10^5^ cells per well, and allowed to adhere for 3 min. Digital videos were recorded for 20 minutes under a phase contrast light microscope (Olympus Lifescience, Tokyo, Japan) equipped with a Cannon digital camera.

### Cell viability assay

Cells were cultured in 96-well plates to ~100% confluent. Cell viability was assessed with the tetrazolium-based CellTiter 96 Aqueous kit (from Promega, Madison, WI, USA) according to the manufacturer’s direction.

### Small GTPase pull-down assay

Cells were cultured to approximately 80% confluence. Before experimentation, cells were washed with serum free medium, and then incubated with vehicle or 10 μM PTPI22 for different for 20 or 40 min. Pull-down assay for GTP-bound Rac1 was performed using a Rac1 Activation Assay Kit from EMD Millipore (Billerica, MA, USA). For western blot detection we used an anti-Rac1 antibody from Cell Signalling Technology (Cat# 2465) (Beverley, MA, USA) in stead of the original antibody provided in the kit.

### Transfection with siRNA

Three sequences of siRNA targeting DOCK180 or Vav2 were purchased from GenePharma (Shanghai, China). Transfection was performed using Lipofectamine RNAiMAX Reagent (Life Technologies, Carlsbad, CA, USA) according to the manufacturer’s protocol. Experiments were carried out 48 hr after transfection. The gene silencing efficiency was determined by western blot.

### Western blot and immunoprecipitation

Total proteins were extracted in cold lysis buffer containing 50 mM Tris, pH 7.5, 2 mM EDTA, 100 mM NaCl, 50 mM NaF, 1% Triton X-100, 1 mM Na_3_VO_4_ and 40 mM β-glycerol phosphate, with added protease inhibitor cocktail (Roche, Mannheim, Germany). For immunoprecipitation, equal amount of protein samples were precleared and incubated with 2 μg of antibody and 20 μl of 50% protein A/G agarose bead slurry (Pierce Biotechnology, Rockford, IL, USA) at 4 °C for overnight with gentle agitation. The beads were washed and boiled in 3× Laemmli buffer. Protein samples were separated by SDS-PAGE and transferred to nitrocellulose membranes. The membranes were developed with HRP-conjugated secondary antibodies and ECL Prime reagents (GE, Piscataway, NJ, USA). Signals were detected with a LAS-4000 luminescent image analyzer (Fujifilm, Stamford, CT, USA). The densitometry analysis was performed using Image-J software (NIH). The following antibodies were used: anti-phospho-tyrosine (#9411), Src (#2110), FAK (#3285), Vav2 (#2848), and p130Cas (#13383) were from Cell Signalling; anti-Src (ab109381) and Crk (ab133581) were from Abcam (Cambridge, UK); anti-paxillin (#05–417) was from EMD Millipore; anti-Tiam1 (AF5038) was from R&D Systems (Minneapolis, MN, USA); anti-DOCK180 (sc-6167) was from Santa Cruz (Dallas, Texas USA).

### Immunofluorescence

Cells cultured on Lab-Tek chamber slides were fixed with paraformaldehyde and rinsed in PBS. Cells were incubated with anti-Crk or anti-DOCK180 antibodies (dilution 1:100) overnight and then with Alexa Fluor 488- or 594-conjugated secondary antibodies (Jackson ImmunoResearch Laboratories, West Grove, PA, USA) at room temperature for 2 hr. Cells were counterstained with DAPI for 15 min. Fluorescent images were taken with the confocal microscope.

### Statistical analysis

All experiments were independently repeated at least three times. Data are presented as mean ± standard error of the mean (S.E.M.). Data analysis was performed with unpaired *t*-test or one-way ANOVA followed by *post hoc* Newman-Keuls test as appropriate. *P* < 0.05 was considered as statistically significant. All tests were two-tailed.

## Additional Information

**How to cite this article**: Wang, Y. *et al*. PTP1B inhibitor promotes endothelial cell motility by activating the DOCK180/Rac1 pathway. *Sci. Rep.* **6**, 24111; doi: 10.1038/srep24111 (2016).

## Supplementary Material

Supplementary Figures

Supplementary Video S1

Supplementary Video S2
