# Supplementary material for: PTP1B inhibitor promotes endothelial cell motility by activating the DOCK180/Rac1 pathway
Source: Sci Rep. 2016 Apr 7;6:24111. doi: 10.1038/srep24111 (PMC4823726; doi:10.1038/srep24111)
Supplement: Supplementary Figures [file srep24111-s1.pdf]

PTP1B inhibitor promotes endothelial cell motility by activating the DOCK180/Rac1 pathway

Authors:

Yuan Wang
Feng Yan
Qing Ye
Xiao Wu
Fan Jiang

Supplementary Figure S1

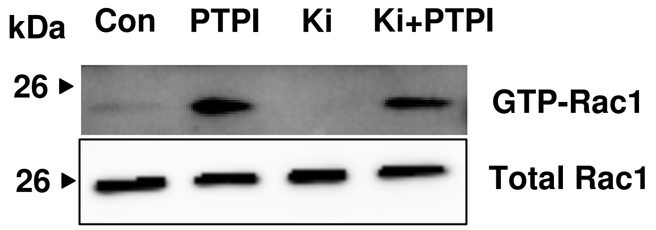

Western blot showing that PTPI22 (10 µM for 20 min) could stimulate Rac1 activation in the presence of the VEGFR2 antagonist Ki8751 (2 nM) in TIME cells. Representative data from 3 experiments.

Supplementary Figure S2

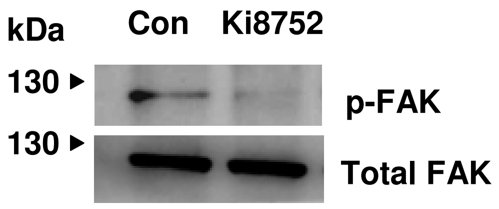

Western blot showing that Ki8751 (2 nM for 60 min) reduced the phosphorylation level of FAK in normal EC. Representative data from 2 independent experiments.

Supplementary Figure S3

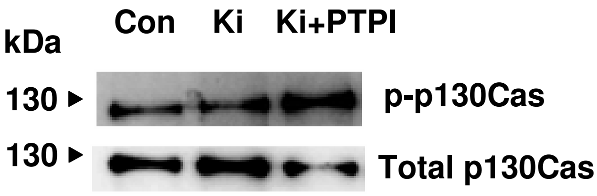

Western blot showing that PTPI22 treatment increased tyrosine phosphorylation of p130Cas in the presence of Ki8751. Representative data from 2 independent experiments.

Supplementary Figure S4

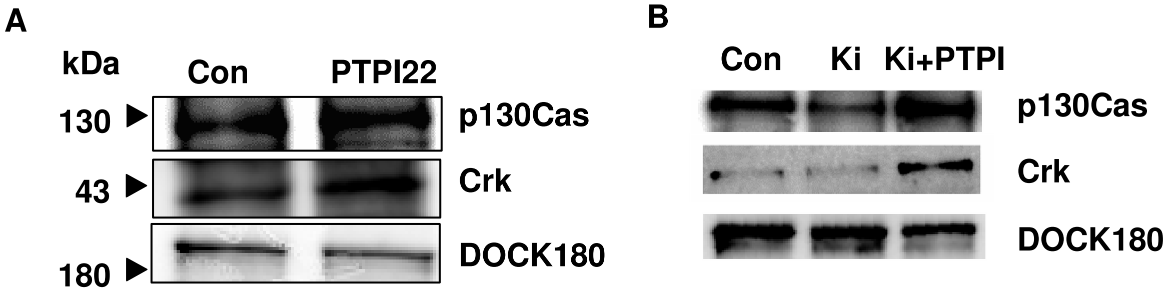

Western blots showing that the bindings of DOCK180 with p130Cas and Crk were increased by PTPI22 in (A) normal ECs and (B) Ki8751-pretreated cells. Samples were immunoprecipitated with anti-DOCK180 and immuno-blotted with antibodies as indicated. Representative data from 2 independent experiments.

Supplementary Figure S5

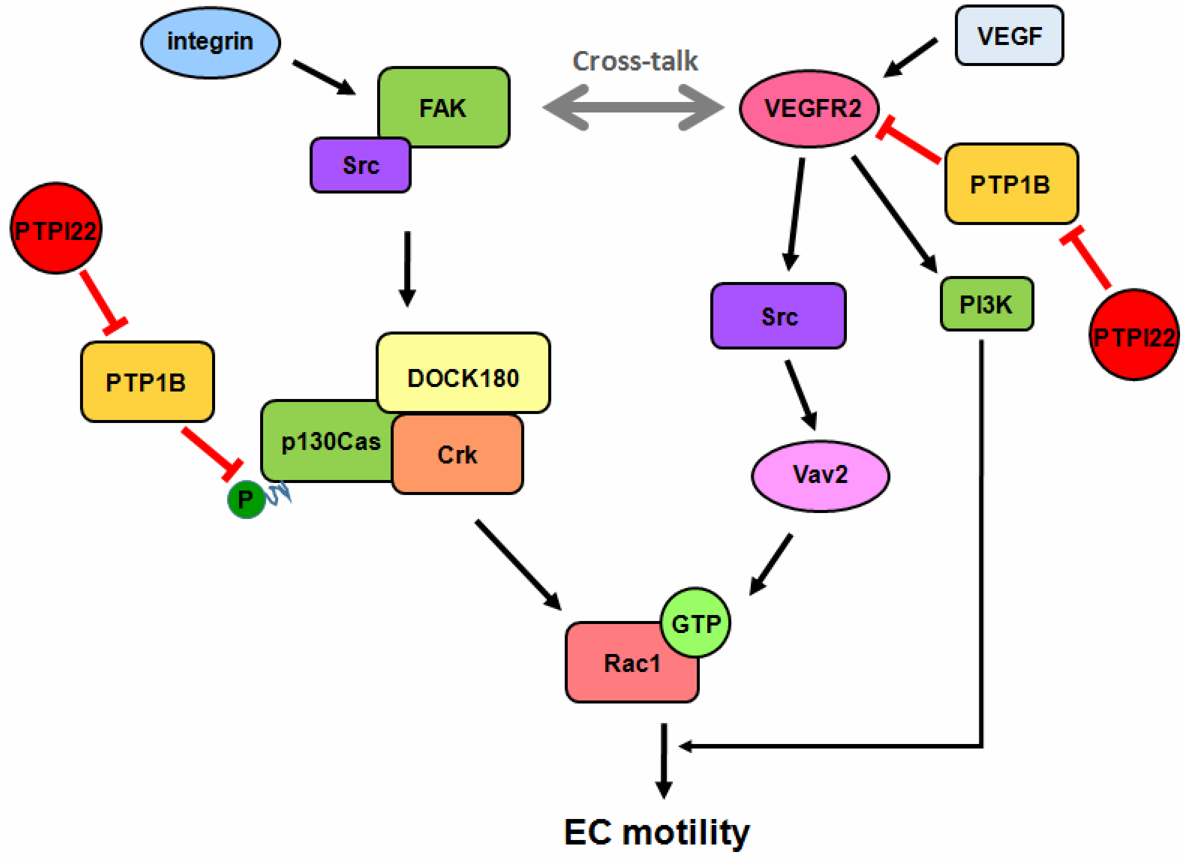

Summary diagram depicting potential signalling pathways and specific effector targets involved in the stimulatory effects of PTP1B inhibitor on EC motility. PI3K, phosphatidylinositol-3 kinase.

Supplementary Videos S1: Dynamic changes of cell spreading process were continuously monitored in untreated TIME cells.

Supplementary Videos S2: Dynamic changes of cell spreading process were continuously monitored in PTPI22-treated TIME cells.
